# Dynamic Dielectrophoresis Model of Multi-Phase Ionic Fluids

**DOI:** 10.1371/journal.pone.0117456

**Published:** 2015-02-20

**Authors:** Ying Yan, Jing Luo, Dan Guo, Shizhu Wen

**Affiliations:** Tsinghua University, State Key Lab of Tribology, Beijing, P. R. China; Massachusetts Institute Of Technology, UNITED STATES

## Abstract

Ionic-based dielectrophoretic microchips have attracted significant attention due to their wide-ranging applications in electro kinetic and biological experiments. In this work, a numerical method is used to simulate the dynamic behaviors of ionic droplets in a microchannel under the effect of dielectrophoresis. When a discrete liquid dielectric is encompassed within a continuous fluid dielectric placed in an electric field, an electric force is produced due to the dielectrophoresis effect. If either or both of the fluids are ionic liquids, the magnitude and even the direction of the force will be changed because the net ionic charge induced by an electric field can affect the polarization degree of the dielectrics. However, using a dielectrophoresis model, assuming ideal dielectrics, results in significant errors. To avoid the inaccuracy caused by the model, this work incorporates the electrode kinetic equation and defines a relationship between the polarization charge and the net ionic charge. According to the simulation conditions presented herein, the electric force obtained in this work has an error exceeding 70% of the actual value if the false effect of net ionic charge is not accounted for, which would result in significant issues in the design and optimization of experimental parameters. Therefore, there is a clear motivation for developing a model adapted to ionic liquids to provide precise control for the dielectrophoresis of multi-phase ionic liquids.

## Introduction

Dielectrophoresis (DEP) is an efficient and precise technique that is widely applied to manipulate droplets or particles in electrolyte based microsystems [1–8]. DEP microdevices have attracted increasing interest from the lab-on-a-chip research community for cell manipulation or drug delivery due to their simple peripheral equipment and easy integration with microchips [9,10]. A high surface area to volume ratio was demonstrated to facilitate a faster reaction time and resulted in high-throughput chemical reactions [11,12]. The independent manipulation of each droplet makes parallel processing and experimentation possible and allows large data sets to be acquired efficiently. The strength of the dielectrophoresis force depends strongly on the medium and the microdroplet’s electrical properties.

Ionic liquids are widely used in electro kinetic experiments and biological experiments because of their conductivity and biocompatibility. Plecis et al studied the ionic fluid transport process in microfluidic systems for the application for a simultaneous preconcentration and separation of biomolecules within simple straight channels [13]. The ionic liquids are applied as surfactants to induce the droplets emulsion in the microchannels [14]. In another effort, ionic liquids have been applied as the strain gauges in a microfluidic pressure sensing system [15]. Ionic liquids are also of great function in AC-electrokinetics (ACEK) method to manipulate fluid flow on electrolyte-based microfluidic systems [16].

The dielectrophoresis manipulation of ionic droplets has been investigated comprehensively using experimental and numerical methods. The microdroplet shape and size, the ion concentration and the frequency of the electric field can influence the dielectrophoresis force to some extent. Gallo-Villanueva, R. C. et al [17] investigated the conductivity of the suspending medium (KH_2_PO_4_), the local electric field, and the gradient of the squared electric field and found that these factors influenced the dielectrophoresis force exerted on polystyrene microspheres. Jung, Y. M. et al [18] investigated the electric charging of a deionized water droplet in a dielectric fluid on the electrode surface and the subsequent electrophoretic motion of the charged droplet. Although the experimental observations and the charging mechanism have not been explained in detail, numerous possibilities have been found for using the electrophoresis of a charged droplet as a new droplet manipulation method [19]. The complicated variation in surface tension and an induced dipole must be understood to predict the motion of the droplet in electro wetting and DEP. Many theoretical [20,21] and numerical [22,23] studies have been performed to explain the underlying physics. However, the effects of ion migration and electrophoretic migration in two-phase flow have not been investigated in detail. According to the analysis above [17,23], the ionic fluid applied in the microchannel had an obvious effect on the flow patterns and on the droplet dynamic behaviors. It is essential to determine how the ions affect the droplets to realize better droplet control.

Numerical simulations regarding dielectrophoresis phenomena have provided significant contribution to the analysis of dynamic behaviors and major influences of the fluids. Luo J. et al [24] simulated the dynamic mechanism of bubbles in a microchannel under the effect of dielectrophoresis. They set up a simulation model that couples the electric field with the flow field. The distributions of the Coulomb force and the electric force on the medium interface were studied, and the characteristics of the force were analyzed in detail. However, in real applications, ionic liquids with different conductivities are used. The pH, ion density and electric field frequency influence the distribution of net charge and charge polarization significantly.

In this work, a numerical method is used to simulate the dynamic behavior of ionic droplets in a microchannel under the effect of dielectrophoresis; the model was developed with the Comsol multiphysics software. The ion concentration of the sodium chloride solution (NaCl) is considered to form a more accurate model. The Navier-Stokes equations are solved using the level set method, which considers the deformable interface between the water droplets and oil. Both the polarization Coulomb force and the dielectrophoresis force are considered the force sources of the Navier-Stokes equations. Using these sources, the Navier-Stokes equations are coupled and solved using the finite element method. In addition, the ion migration and the electrophoretic migration are considered in the dielectrophoresis force calculation. The electric field force of the droplet and the deformation of the droplet are investigated, and the effect of the net charge on the electric field force is considered. The goal of this work is to further develop means for the precise manipulation of microdroplets and microparticles.

## Materials and Methods

### Theory

Generally, for spherical droplets suspended in a medium in either an AC or DC electric field, there are three types of forces that can affect the dynamic behaviors of the droplets: the Coulomb force, the DEP force, and the interfacial tension force. The DEP force and electric field force are generated by the non-uniform electric field and the permittivity of the two-phase flow. Modeling the electric field is performed using the electric potential ***V***, as follows [23]:

E=−∇V (2-1)

$$\nabla \cdot (\varepsilon \cdot E)=\rho_{e}$$ (2-2)

where *E* is the electric field intensity, *ε* is the permittivity and *ρ*_*e*_ is the free charge density. According to dielectrophoresis theory, when a dielectric drop is polarized by an electric field in a continuous dielectric fluid, electric stress is generated by the electric dipole moment and is given by the Maxwell stress tensor [23],

$$T_{e}=\varepsilon \cdot E\cdot E-\varepsilon \left(E\cdot E\right)\cdot \text{I}/2$$ (2-3)

where I is the identity tensor. This expression can also be expressed in terms of force density [25]:

$$f_{e}=\frac{1}{2}\nabla \left[E^{2}\cdot \varepsilon \right]-\frac{1}{2}E^{2}\cdot \nabla \varepsilon$$ (2-4)

where the first term is the Coulomb force density generated by the polarization charge and the second term is the dielectrophoretic force density.

However, if there are ions present in the fluid, ion migration cannot be neglected. The transport and mass balance of ions at low concentrations in the electric field are given by the electrokinetic flow equations without reactions [26,27]:

$$\frac{\partial c_{i}}{\partial t}+\nabla \cdot \left(-D_{i}\cdot \nabla c_{i}-z_{i}\cdot m_{i}\cdot F\cdot c_{i}\cdot \nabla V+c_{i}\cdot u\right)=0$$ (2-5)

where *i* represents ionic species in the solution, *c* is the concentration of ion, *F* is the Faraday constant (96485C/mol), *m* is mobility, ***u*** is the fluid velocity, *z* is the ion valency. The diffusion coefficient, *D*, is

$$D_{i}=R\cdot T\cdot m_{i}/\left|z_{i}\right|$$ (2-6)

where *R* is the gas constant and *T* is the absolute temperature. The net charge density is defined as

$$\rho_{n}=F\cdot \sum_{i=1}^{n}z_{i}\cdot c_{i}$$ (2-7)

In fact, *ρ*_*e*_ in equ.(2–2) is equal to *ρ*_*n*_. The total charge density at the interface between the two fluids, assuming charge due strictly to polarization, can be given by

$$\rho_{p}=-\nabla \cdot \left(\varepsilon -\varepsilon_{0}\right)\cdot E\left(n\right)$$ (2-8)

If the interface charge consists entirely of net charge equivalent to a metal conductor in a direct electric field in electrostatic equilibrium, it can be given by

$$\rho_{s}=\nabla \varepsilon_{0}\cdot E\left(n\right)$$ (2-9)

where *ε*_*0*_ is the vacuum permittivity. The total charge density should fall between these bounds, i.e., between *ρ*_*p*_ and *ρ*_*s*_. With regards to the value of the net charge density, the electrolyte can be considered an equivalent dielectric with infinite permittivity. The relative permittivity of the water and the oil are 80 and 2, respectively; thus, according to preliminary calculations, the tolerance between *ρ*_*p*_ and *ρ*_*s*_ can be ignored. In this work, we consider the net charge to replace the effect of polarization charge so that the total charge density is equal to *ρ*_*p*_. Thus the actual polarization charge density is

$$\rho_{pa}=\rho_{p}-\rho_{s}$$ (2-10)

The net charge does not change the total charge, so the Coulomb force density is not affected; however, the dielectrophoretic force decreases with incomplete polarization. Therefore, equ.(2–4) is accordingly revised:

$$f_{e}=E\cdot \rho_{p}+\frac{\rho_{pa}}{\rho_{p}}\left(-\frac{1}{2}E^{2}\cdot \nabla \varepsilon \right)$$ (2-11)

The flow field is formulated by the Navier-Stokes equations. Gravity is usually ignored in micro-devices, so the velocity field for the Newtonian fluid can be written as [24]

$$\rho \cdot \partial u/\partial t+\rho u\cdot \nabla u=-\nabla p+\mu \nabla^{2}u+f_{e}+f_{t}$$ (2-12)

where the interfacial tension force and electric field force are

$$f_{e}=E\cdot \rho_{\text{e}}+\rho_{\text{p}}/\rho_{\text{e}}\cdot \left(-E^{2}/2\cdot \nabla \varepsilon \right)$$ (2-13)

$$f_{t}=\sigma \cdot k\cdot \delta \cdot n$$ (2-14)

where *σ* is the interfacial tension coefficient, *k* is the curvature of interface and *δ* is the Dirac delta function. Other forces, such as the double layer repulsion, are not considered here. Mass conservation was considered in compressible form:

∇⋅(ρu)=0 (2-15)

To approximate the real physical interface and satisfy the continuity of solutions, the level set method was applied to represent the moving interface using a fixed mesh. The conservative level set method for the motion of interfaces is as follows [24]:

$$\frac{\partial \varphi }{\partial t}+\nabla \cdot \left(u\varphi \right)=\gamma \cdot \nabla \cdot \left(\lambda /2\cdot \nabla \varphi -\varphi \left(1-\varphi \right)\frac{\nabla \varphi }{\left|\nabla \varphi \right|}\right)$$ (2-16)

where *λ* is the interface thickness parameter and *γ* is the reinitialization parameter. The interface domain is represented by a certain level set of a globally defined function *φ*, which is a smooth step function equal to 0 in one domain and 1 in the other. Across the interface domain, *φ* transitions smoothly from 0 to 1 in the form of a high-order curve, and the 0.5 contour of *φ* defines the interface. In this work, the density *ρ*, the viscosity μ, the concentration of ions and the permittivity of fluids are expressed by

$$\rho =\rho_{w}+\varphi \cdot \left(\rho_{o}-\rho_{s}\right)$$ (2-17)

$$c=c_{w}+\varphi \cdot \left(c_{o}-c_{s}\right)$$ (2-18)

$$\mu =\mu_{w}+\varphi \cdot \left(\mu_{o}-\mu_{s}\right)$$ (2-19)

$$\varepsilon =\varepsilon_{w}+\varphi \cdot \left(\varepsilon_{o}-\varepsilon_{s}\right)$$ (2-20)

where the subscripts *o* and *s* represent the oil and the solution, respectively.

### Model and parameters

The density and viscosity of the solution and the oil are 1000 kg/m^3^, 0.001 Pa·s and 780 kg/m^3^, 0.003 Pa·s, respectively. The interfacial tension coefficient is 0.01 N/m. The concentrations of positive ions Na^+^ and negative ions Cl^-^ are 0.1 mol/m^3^, which are similar to that of fluids used in AC electrokinetic microdevices used in a micropump, and the charge number is +1/-1. To simplify the calculation, the electrophoretic migration rate is set as a constant: 5.4×10^-13^ s•mol/kg (ion migration rate 5.2×10^-8^ m^2^ / (V•s)). The temperature is 20°C, and the diffusion coefficient is 1.3×10^-9^ m^2^/s. The conductivity of this model is 10^-3^ S/m[28]. In order to figure out the influence of ion concentration, three groups of calculation models with different ion concentration of 0 mol/m^3^,0.01 mol/m^3^ and 0.1 mol/m^3^ are designed. The conductivities of these models are 0 S/m, 10^-4^ S/m and 10^-3^ S/m respectively. The frequency is set as 10^4^ Hz and the voltage applied on the electrode is $400\sqrt{2}\cos\left(2\times \pi \times 10000t\right)$. There are no chemical reactions in the model, and R is equal to 0. The parameters are defined according to basic physical and chemical properties (Table 1). This research focused on the effect of ion concentration on electric field force. Though the joule heating effect is also a key point in the ionic droplet based work [29], it is not considered in the calculation.

**Table 1:** The parameters used in the simulation work.

**Parameter**	**Value**	**Parameter**	**Value**
**Solution density *ρ***_***s***_	1000 kg/m^3^	**Electrophoretic migration rate**	5.4×10^-13^ s·mol/kg
**Viscosity *μ***_***s***_	0.001 Pa·s	**Frequency**	10^4^ Hz
**Permittivity *ε***_***s***_	80		
**Solution density *ρ***_***o***_	780 kg/m^3^	**Conductivity**	10^-3^ S/m
**Viscosity *μ***_***o***_	0.003 Pa·s	**Voltage effective value**	400V
**Permittivity *ε***_***o***_	20		
**Interfacial tension coefficient *σ***	0.01 N/m	**Temperature**	20°C
**Ion Concentration *c*** 0 mol/m^3^ 0.01 mol/m^3^ 0.1 mol/m^3^			

**Fig 1:**
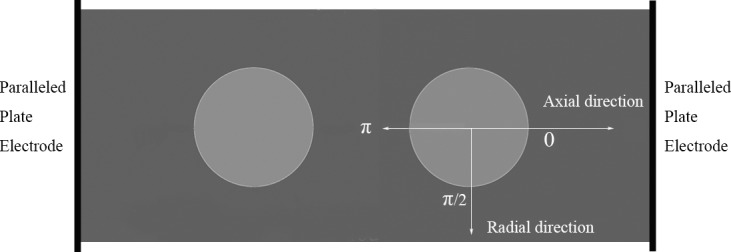
The schematic of the model about the simulation.

As shown in Fig. 1, the simulation consists of a cylindrical microchannel and two solution droplets suspended in the microchannel. Two solution droplets are immersed in the oil at the axial line of the cylinder channel, and the radius of each droplet is 20 μm, as shown in Fig. 1; the distance between two droplets is equal to the diameter of the droplets. Also, the regions along the axial direction are defined as the “polar region” and the other parts are “middle region”. The thickness of the interface domain is set to 1 μm. The positive direction is defined as the pointing from left to right and from top to bottom. The phase angle is defined according to the clockwise direction. The cylindrical channel is 400 μm in length and 80 μm in diameter, and the pressures at the outlet and inlet are set to zero. Two planar electrodes attached at the ends of the channel are considered to be infinite, and the channel wall is modelled with a symmetric boundary condition for the electric field equations to create a uniform electric field far away from the droplets.

To achieve precise solutions but avoid an overly large number of meshes from exhausting the computation time, the axisymmetric geometry model is adopted. FEM is used to simultaneously solve the time-dependent, second-order partial differential equations in two dimensions, as expressed in their weak (integral) forms, using the weighted residual approach. The electric field equations, the flow field equations and the electrokinetic flow equations are coupled by the function variable, *φ*. Both the velocity and the potential feature second-order accuracy, but a linear element for the pressure is used for the sake of calculation stabilization. The curvature of the interface domain is calculated using the finite difference method. The second-order implicit backward differencing method is used for the time integration. The solution domain is meshed with a non-uniform triangle mesh using the advancing front method, and a higher resolution is applied at the droplet domain, particularly at the initial interface region. Due to the requirement of level set method, the maximum mesh size is less than or equal to the defined thickness to ensure at least one layer of mesh in the transition region. The minimum size of the element is 1.2×10^-7^m and the number of the mesh elements is 103367. It takes about 7670s for one calculation model on the compute server (Dell X5660,Texas, USA). The reinitialization parameter, *γ*, is a time-varying function, and its approximate value can be represented by the fluid speed. However, the speed could not be known in advance, so the proper value is determined during an initial calculation with a time period of 10^-8^ s; *γ* is subsequently modified using the calculated speed in the subsequent time steps. The absolute tolerance for the variables is set to 1×10^-6^.

## Results and Discussion

### Droplet transportation and deformation in the DEP microdevices

Generally, the ion concentration influences both the electric field force and the dielectrophoresis force. If the electric field is not considered, the positive and negative ions uniformly distribute in the solution, and the solution does not show an electrical property. However, when introducing an alternating electric field, the ions move towards the electrodes. Therefore, the local concentration of the solution is altered and results in net charge. With variations in the electric field, the direction of the charge transmission and the electrical property of the net charge change. The local electric field distribution is also altered.

To study the characteristics of the charge, the role of the electric field force at the interface is not considered during the first two periods, so there is no deformation of the interface area, and the ions outside the interface region achieve a dynamic equilibrium state under the influence of the electric field force. This method also avoids the influence of an initial state of electrically neutral ions in a comparative analysis of the results. This state is then regarded as the initial value to be marked as the 0^th^ hour. The calculation subsequently considers the electric field force.

Due to the existence of Na^+^ and Cl^-^ ions, the droplet is a weak conductive solution. It is known that the net charge moves to the surface such that the electric field of the internal part is 0, according to the physical properties of a conductor in an electric field. In this model, Na+ and Cl^-^ ions move toward the respective poles under the effect of electric field. Although the positive and negative ions move in opposite directions, the balance of the internal charge is unaffected. In fact, the net charge is only distributed within the interface domain. Due to the rate of ion transmission related to the ion nature and the strength of the electric field, the distribution of net charge contains the relaxation process. With an AC field, positive and negative net charge appear alternately and coexistent within the local area. Based on the results, it is concluded that the net charge strength reduced and that the peak value of the net charge decreased with a decrease in the electric field. Alternatively, the net charge with an opposite electric property appeared, and the contribution of the net charge to increasing and transforming the electric field gradually increased.

**Fig 2:**
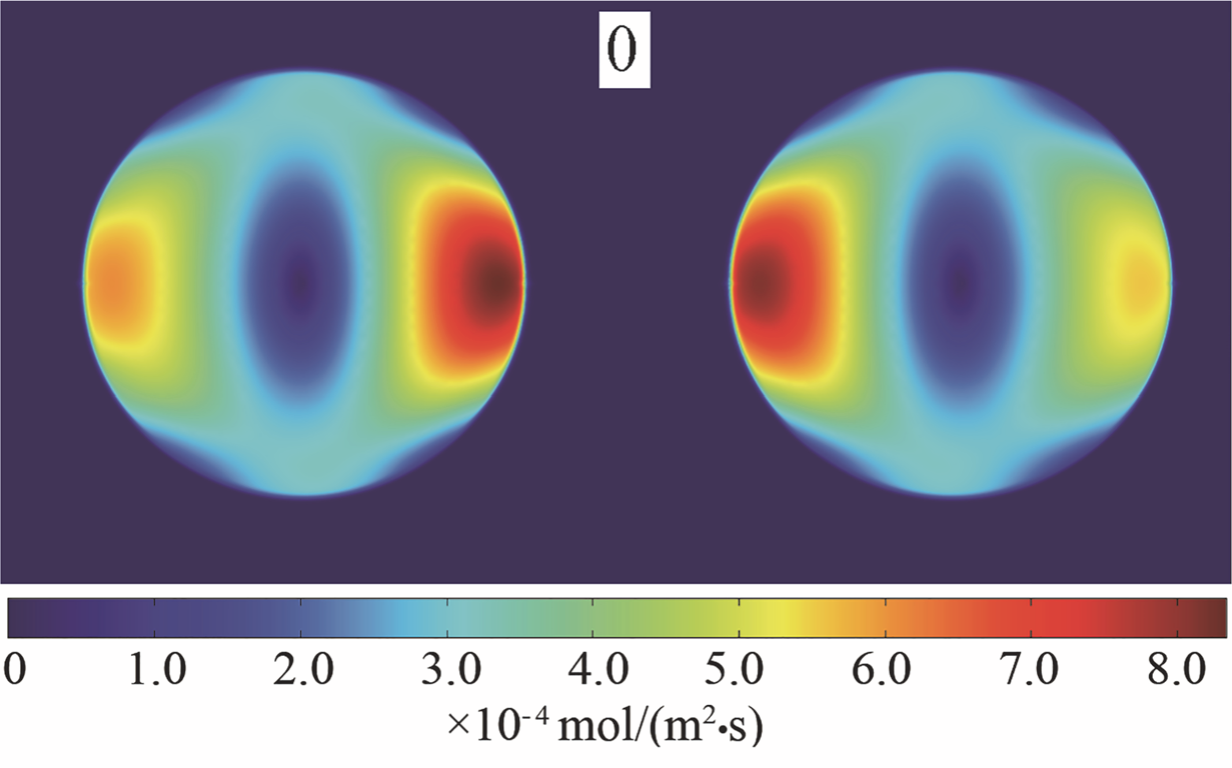
The deformation and ion distribution of the droplets. (0T)

**Fig 3:**
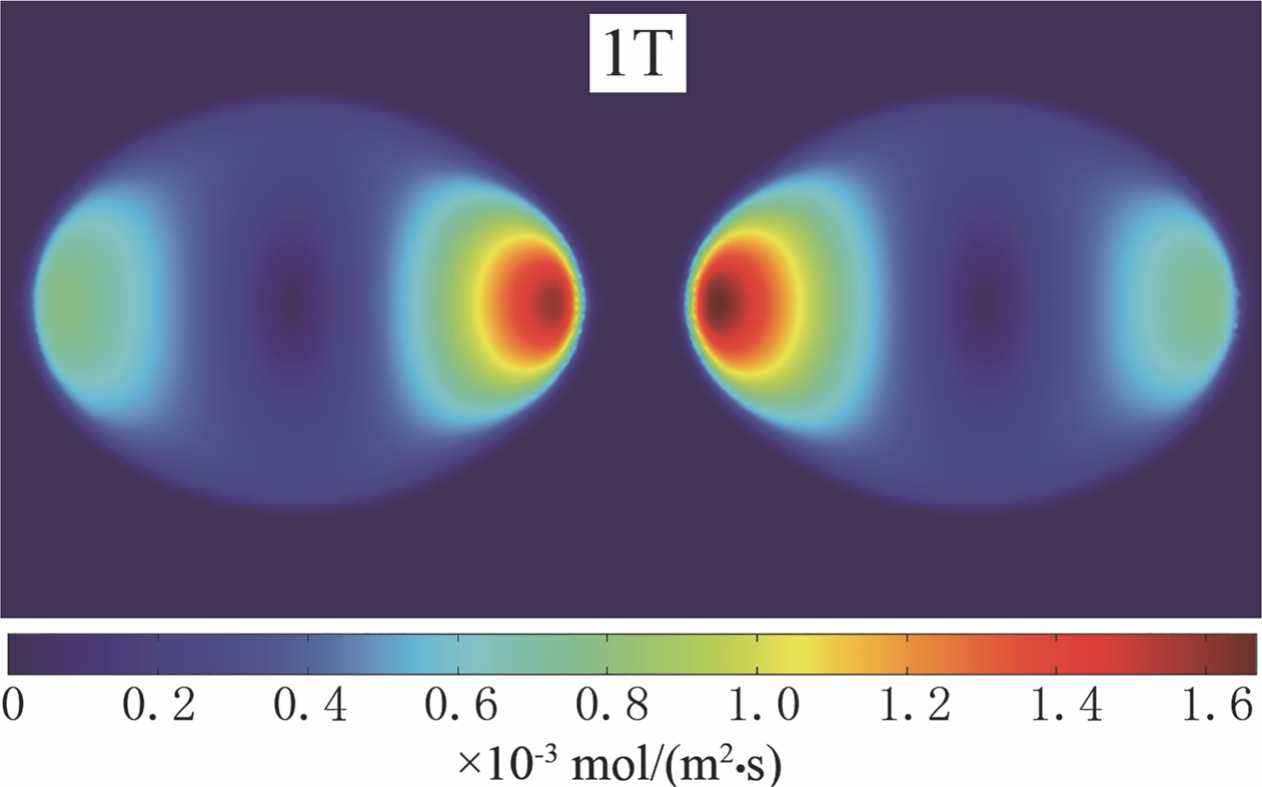
The deformation and ion distribution of the droplets. (1T)

**Fig 4:**
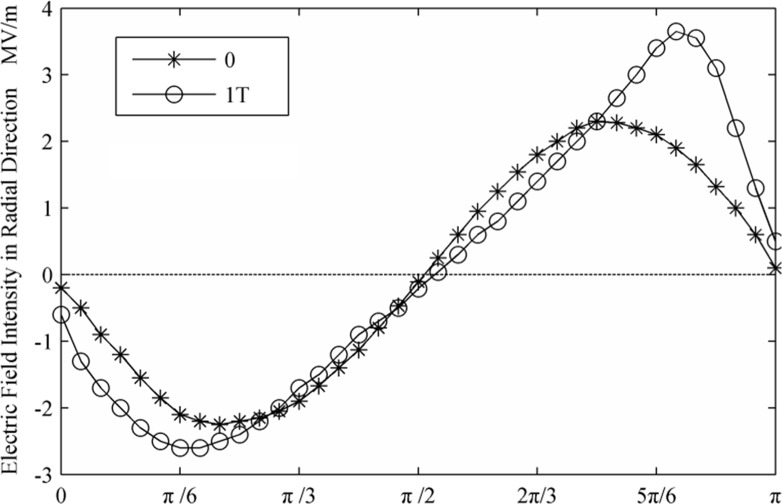
Electric field intensity in radial direction.

**Fig 5:**
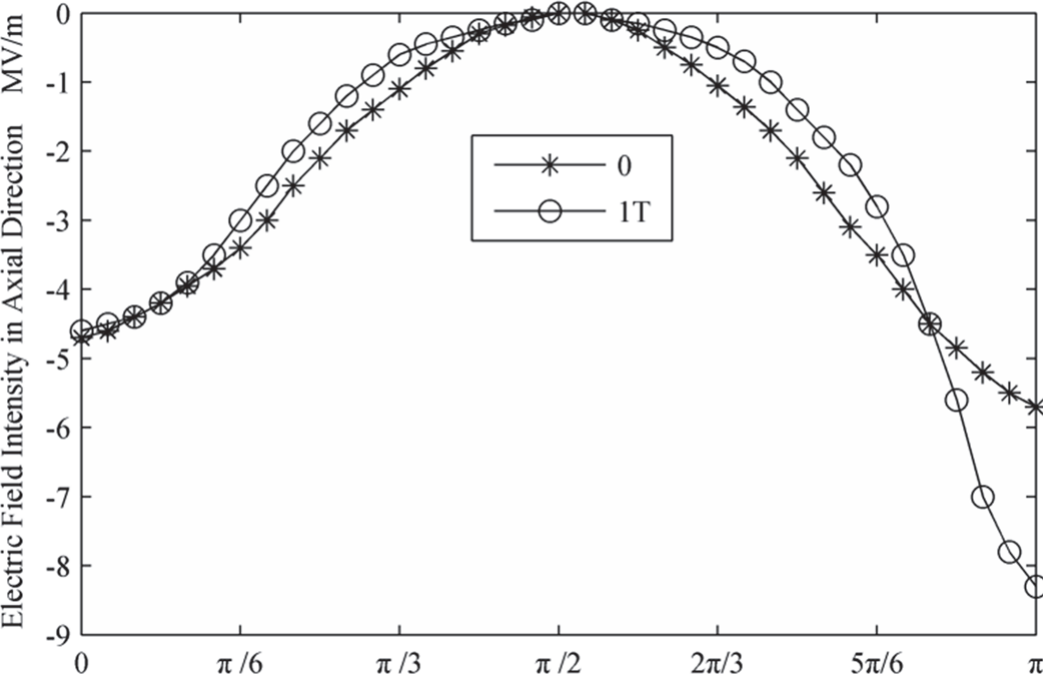
Electric field intensity in axial direction.

Fig. 2 and Fig. 3 displays the deformation of the droplets and the ion distributions under the effect of an electric field. The initial and altered forms of the droplet after a period are shown (0 and 1T). It is evident that droplets are stretched along the axial direction with the electric field force. The curvature of the two poles increased, and the middle region shrank. Thus, the strength of the electric field between the regions along the axial direction of the droplets increased. The ion distribution reflects the electric field intensity; this study shows that the axial direction and the interface area reached higher electric field intensity. According to the distribution of the electric field along the flowing channel radial axial direction and the axial direction, as shown in Fig. 4 and Fig. 5, it can be concluded that the strength of the electric field increased rapidly together with a decrease in the distance between droplets. After a period of deformation, both the curvature of the two poles and the electric field strength increased. The electric field strength decreased with a decrease in the curvature of the middle region. At the same time, the location of the maximal electric field strength transitioned from the π/4 region to the π/6 region.

**Fig 6:**
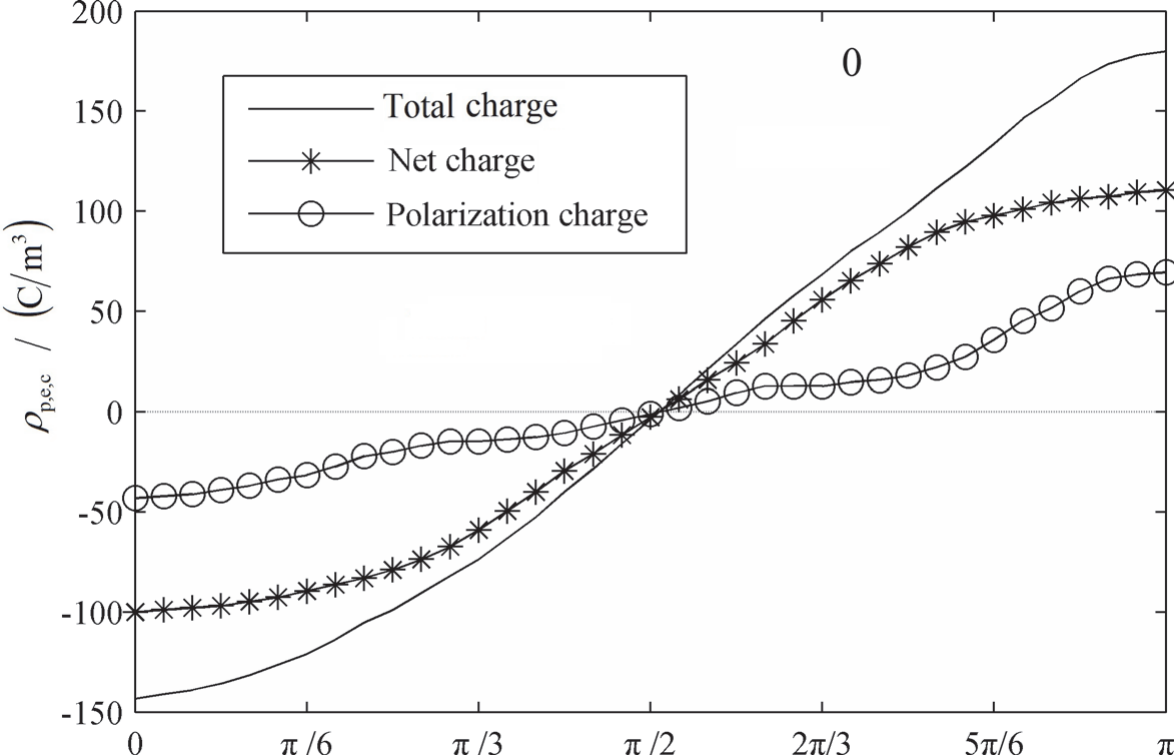
Total charge, net charge and Polarization net charge distribution around the interface. (0T)

**Fig 7:**
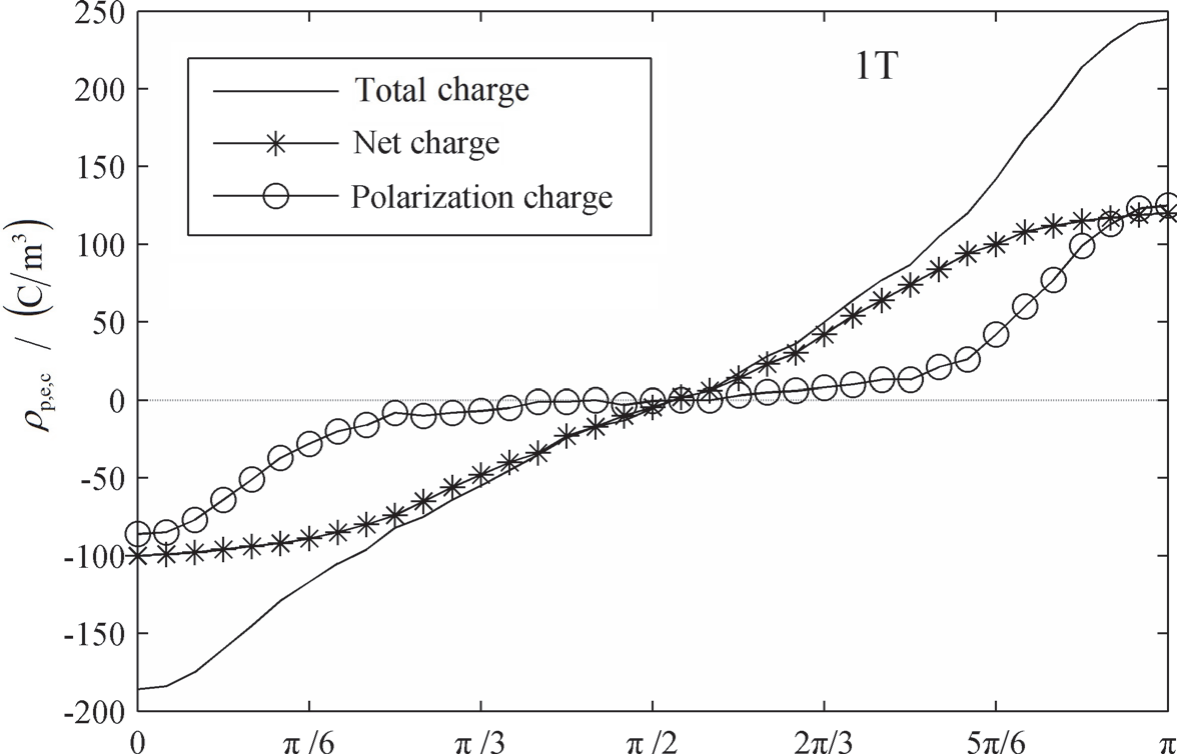
Total charge, net charge and Polarization net charge distribution around the interface. (1T)

In Fig. 6 and Fig. 7 the distribution of net charge, ion net charge and polarization charge at the initial condition and a cycle after that are shown. The positive and negative ions diffusion flux is relative small and these results in a larger relaxation time. Therefore, the positive and negative ions cannot achieve instantaneous equilibrium when exposed to an AC electric field and there would be polarization charge generated around interface. It can be figured out that the distribution curve of ion net charge is similar with the cosinusoid, the peak value occurred at the moment phase angle equals to 0 and π. At the initial moment, the net charge was about half amount of the total charge. If the net charge were treated as the polarization charge, the dielectrophoresis force would be as high as 70% especially when the ion concentration was high. The polarization charge are mainly distributed in the polar regions of the droplet, the equatorial region can be ignored approximately. As the droplets are stretched along the axial direction, ion net charge distribution is stable and without any change. However, the electric field intensity of the polar region increased and the amount of polarization charge would change from 30% to about 50% of the amount of total charge. In the case of droplet tensile deformation is not too severe, the proportion of ions net charge is always higher than polarization charge. The amount of ions net charge of the region away from the polar region almost equals to the total charge and this will significantly affect the droplet interface dielectric polarization and dielectrophoresis force distribution.

**Fig 8:**
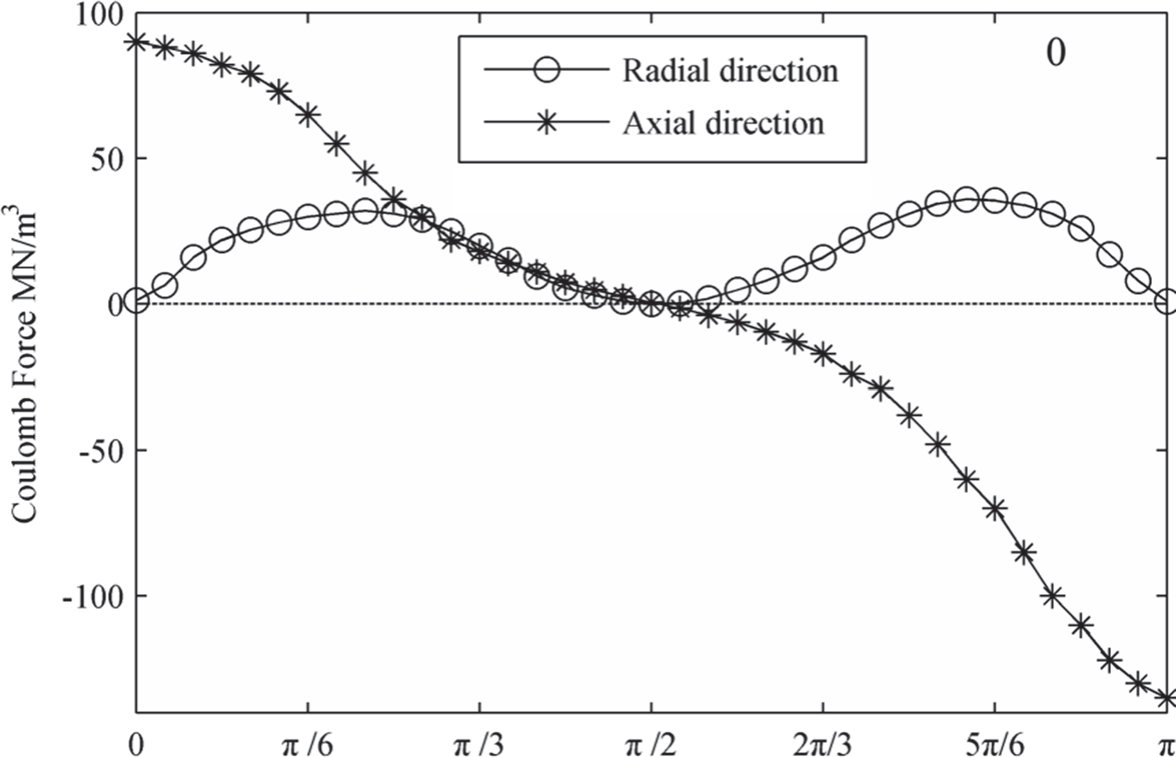
The distribution of the Coulomb force on the interface. (0T)

**Fig 9:**
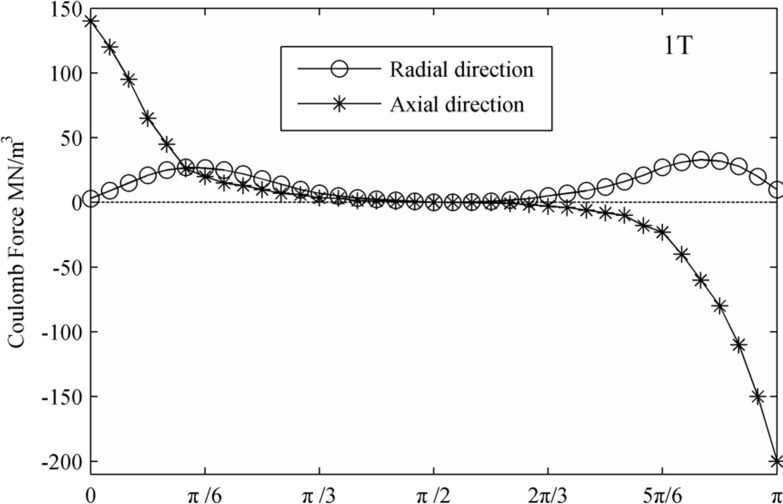
The distribution of the Coulomb force on the interface. (1T)

Fig. 8 and Fig. 9 display the Coulomb force distribution at the interface at the initial condition and a period after that. Because the Coulomb force is the product of the electric field strength and electric charge, its charge distribution is a result of contributions from both the electric field and charge. Generally, the Coulomb force along the axial direction is larger than that in the radial direction. Additionally, with the deformation of the droplets, charge concentration increases in the region along the axial direction of the droplet. As a result, the amplitude of the axial component of the Coulomb increased by approximately 50%.

**Fig 10:**
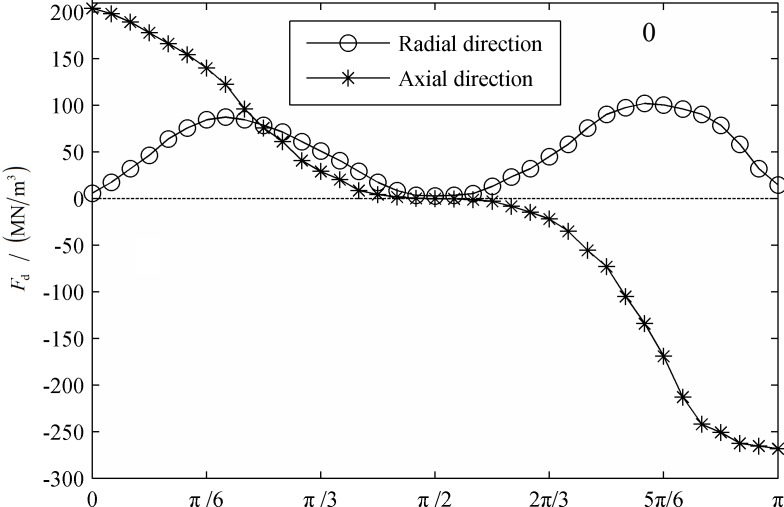
The distribution of the dielectrophoretic force on the interface. (0T)

**Fig 11:**
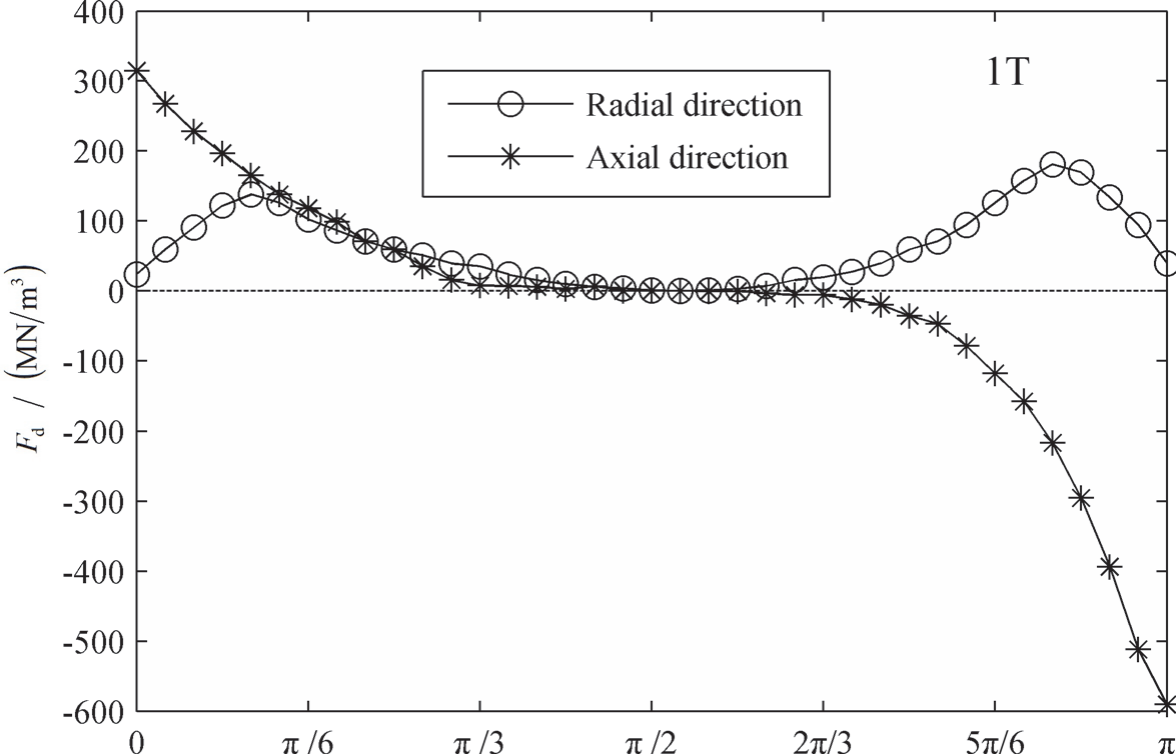
The distribution of the dielectrophoretic force on the interface. (1T)

Fig. 10 and Fig. 11 display the distribution of the dielectrophoretic force at the interface at the initial condition and a period after that. It is known that the dielectrophoresis force depends on the change in the electric field intensity and polarization. In this model, the distribution of the dielectrophoresis force is similar to that of the Coulomb force, but twice the value. The two droplets expanded and moved toward each other. The intensity of the polarized charges increased, as did the dielectrophoresis force. Additionally, both the axial and radial amplitude components of the dielectrophoresis force increased by approximately 50% during this period.

### Influence of Ion concentration on droplet dynamic behaviors

**Fig 12:**
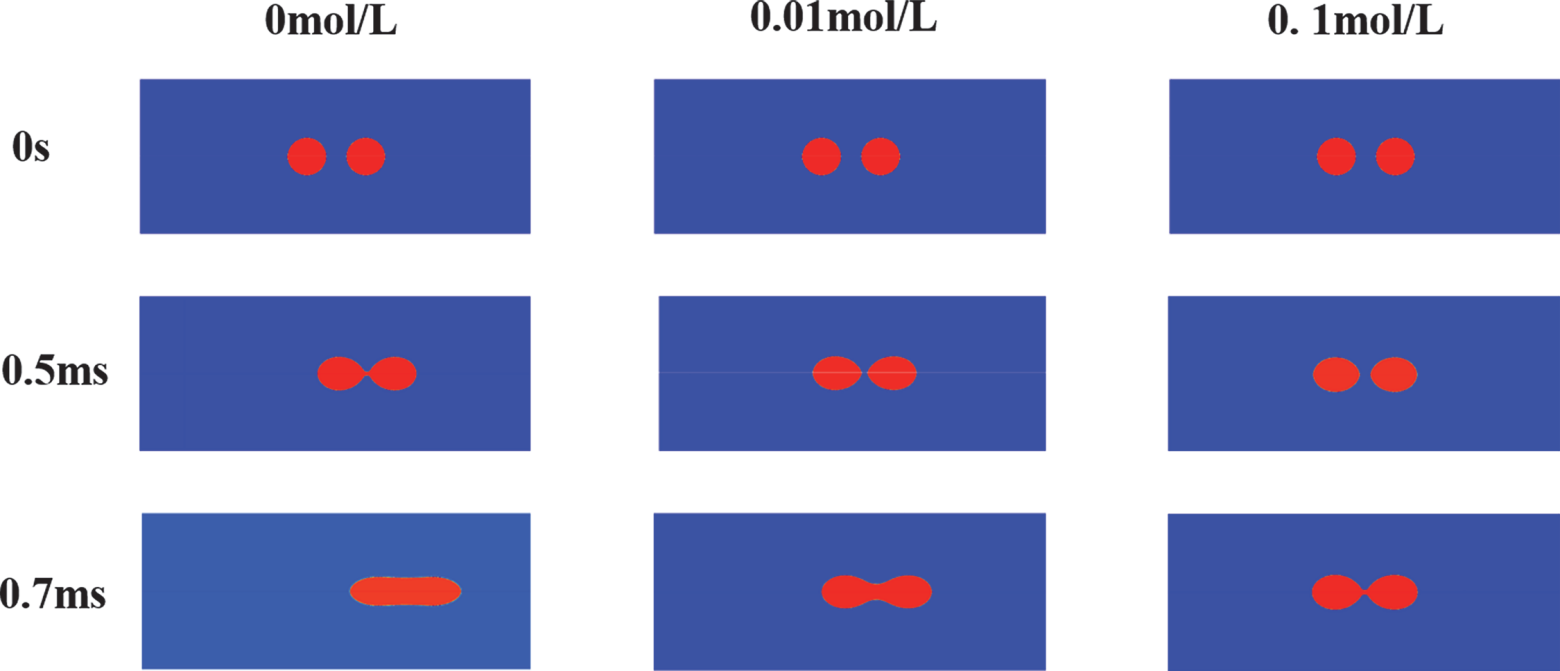
Droplets deformed process in microchannels with different ion concentration.

There were two sodium chloride droplets suspended in the microchannel, which was embedded with electrodes. Under the non-uniform AC electric field, the droplets were simultaneously deformed and moved. As displayed in Fig. 12, it was determined that the droplet dynamic behavior was affected by the ion concentration. Initially, the two droplets were stable before beginning to deform and move with the dielectrophoresis and Coulomb forces. When the concentration was 0 mol/L, the two droplets could contact with each other and began to merge at 0.5 ms. However, when the concentration was increased to 0.01 mol/L, the two droplets maintained separation. At a concentration of 0.1 mol/L the separation distance was greater than that observed for the other two situations. The average velocities of the droplets were studied to elucidate the droplet merger process.

**Fig 13:**
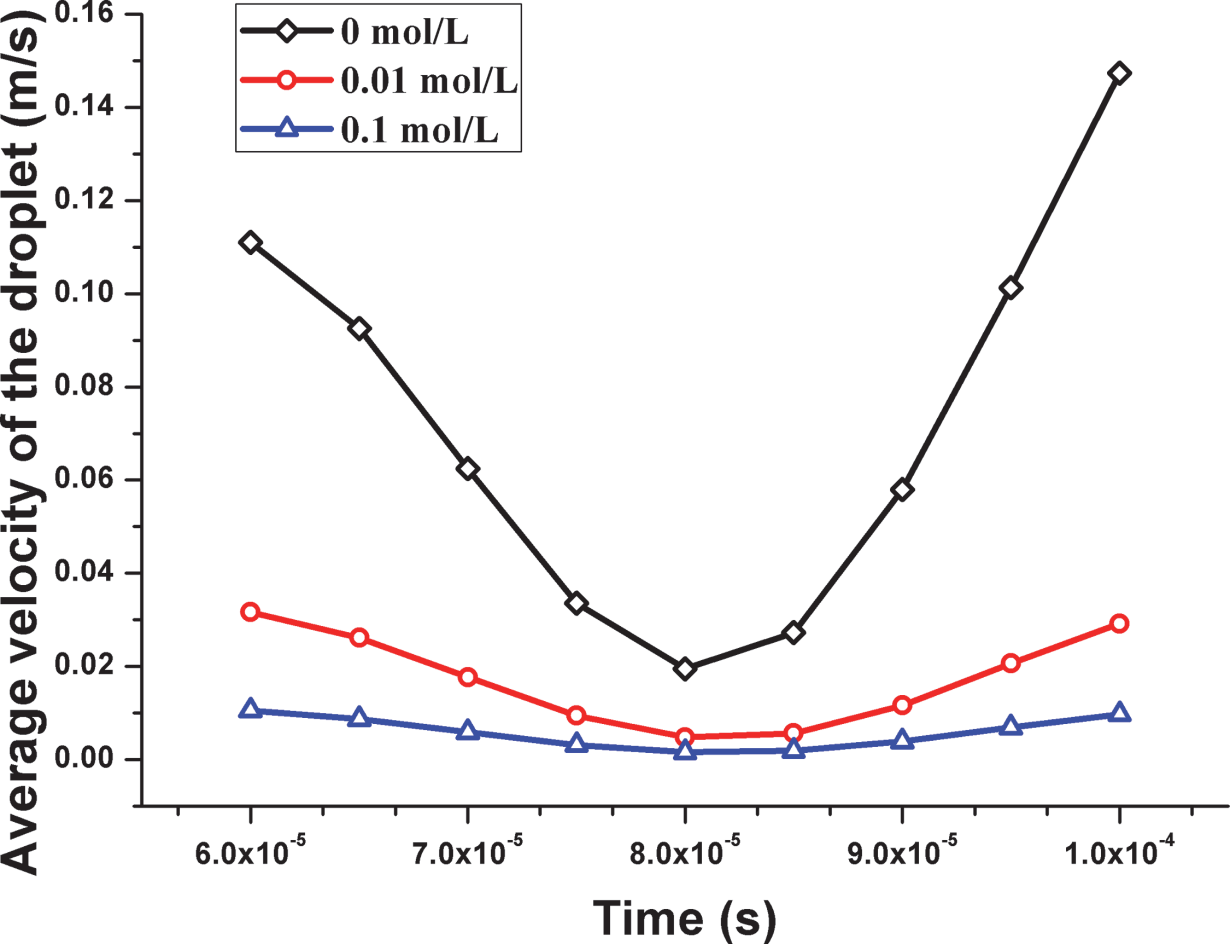
Average velocity of the droplets in the microchannel with different ion concentration.

Under the influence of an AC electric field, the average velocities of the droplet changed with the time. In 2014, Gallo-Villanueva, R. C. et al. determined that the ion distribution influenced the dielectrophoresis force and that the dielectrophoresis force may decrease under certain conditions. As shown in Fig. 13, the average velocity of the droplet on the left decreased with an increase in ion concentration. The same velocity decrease was observed for the droplet on the right before the two droplets began to merge. In general, the velocity of a droplet with a 0 mol/L concentration is 0.72 m/s, which is four times that found for a concentration of 0.01 mol/L. When the concentration was increased to 0.1 mol/L, the average velocity decreased to approximately 0.0058 m/s. After the droplets merged with each other, a new droplet was formed. Subsequently, the ions would redistribute around the interface of the new droplets, and both the dielectrophoresis and Coulomb forces were influenced.

**Fig 14:**
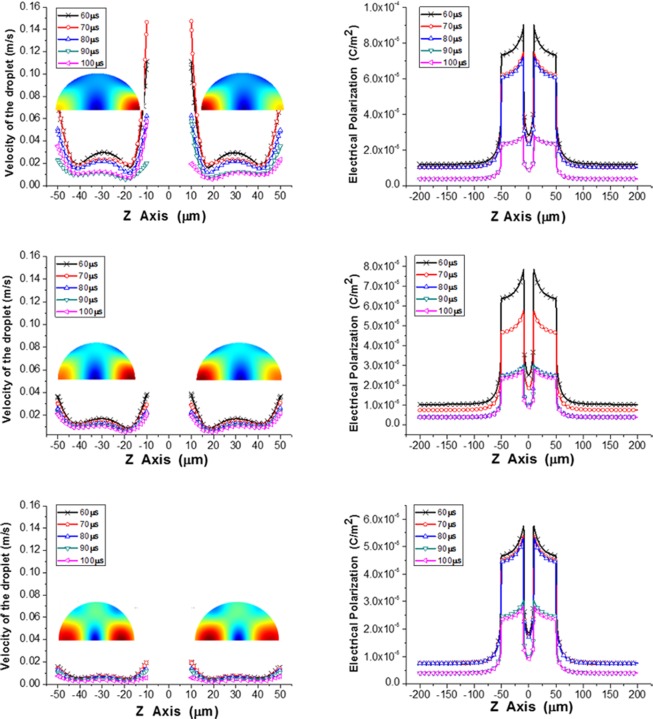
Velocity and Polarization of the microdroplets with different ion concentration.

The velocity and polarization of the microdroplets with different ion concentration are shown in Fig. 14. The z axis is the axial direction of the microchannel, as shown in Fig. 1. The only difference among these three groups of models is the ion concentration. It is evident that with an increase in ion concentration, the velocity of the droplet decreased simultaneously. As previously mentioned, the velocity of the droplets was primarily determined by the Coulomb and dielectrophoresis forces. Because the velocities of different parts of the droplet differed with each other, the droplets were stretched axially. In this work, ion transit was considered, and the charge polarization was consequently affected. When the ion concentration was 0 mol/L, the electric polarization was symmetric about the radial direction. However, if the ion concentration was increased to 0.01 mol/L or 0.1 mol/L, the polarization changed as well.

Because of the relaxation process of the positive and negative ions that occurred when exposed to an AC electric field, the net charges can coexist with each other on the interfacial area. The net charges moved toward the wall alternatively and ultimately disappeared. The net charges changed according to the intensity of the electric field. The frequency of this process is consistent with the electric field. The emergence of net charge ions weakened the polarization strength of fluid medium and the dielectrophoresis force. When the droplets in the electric field stretched, the distance was reduced and the curvature of the poles was increased. As a result, the electric field strength increased rapidly, as did the polarization charge density. Furthermore, both the Coulomb and dielectrophoresis forces increased. The density of the ion net charge remained almost the same, although there was an increase in the electric field strength and changes to the local droplet boundary curvature. Although the dielectrophoresis force appears due to a gradient within the medium, it is produced by the polar moment of the medium and is closely related to the polarization strength. The density of polarization charge increases when the droplets stretch such that they are in close proximity. Accordingly, the medium polarization is strengthened, and the interface electrophoresis force also increases rapidly.

## Conclusions

This study investigated in detail the distribution state of an ion solution medium and the influence of droplet dynamic behaviors under the effects of an AC electric field. Ions are transmitted when exposed to the electric field, and net charges are produced. The net charges not only affect the polarization strength of the medium but also influence the electric field force. The directional migration of positive and negative ions change the charge balance of the solution. Net charges position themselves in accordance with the concentration difference between positive and negative ions. Because the net charge is equivalent to polarization charge in terms of its impact on the electric field, it will enhance or weaken the polarization according to its location. Furthermore, because the impact of net charge and polarization charge are essentially equivalent, there is little influence on the electric field strength and the Coulomb force; however, the effect on the dielectrophoresis force is significant because the dielectrophoresis force is produced by the polarization dipole moment. According to the results, an increase in ion concentration leads to a small dielectrophoresis force, and the droplet motion slows.

Dielectrophoresis in ionic-based microdevices was investigated via numerical simulation. The influence of ion concentration on droplet dynamic behavior was analyzed to determine how to control ionic droplets under the influence of a non-uniform AC electric field. The dynamic behaviors of the microdroplets can be controlled more precisely with the help of an ionic fluid. Dielectrophoresis is a precise and efficient method to control the droplets or particles in microfluidic devices. This simulation work considered the effect of an ionic fluid that is commonly used in biological or chemical experiments, which may contribute to the design of more precise and efficient microchip operation.
